# The Hospital. Nursing Mirror

**Published:** 1898-06-18

**Authors:** 


"The Hospital" Nursing Mirror.

Being the Nursing Section of "The Hospital."

[Contributions for this Section of "The Hospital" should be addressed to the Editor, The Hospital, 28 & 29, Southampton Street, Strand, London, W.C., and should have the word "Nursing" plainly written in left-hand top corner of the envelope.]

### News from the Nursing World.

### PRINCESS CHRISTIAN AT SOHO.

On the afternoon of June 10th the Princess Christian visited the Hospital for Women, Soho Square, and presented the certificates of the St. John Ambulance Association to nine nurse belonging to the hospital.

### THE HOSPITAL OF ST. JOHN OF JERUSALEM.

The Prince of Wales as Grand Prior of the Order of the Hospital of St. John of Jerusalem has recommended four ladies to be Serving Sisters of this Order in recognition of their services during the recent plague epidemic in India. Three of the ladies are the superiors of convents in and near Bombay; the fourth is Mrs. Mabel Allen, now residing in North Kensington, whose sphere of work was the Civil Hospital at Kurrachee. The appointments have recently been notified by the Government of India.

### WOMEN FOR MILITARY DUTY IN AMERICA.

Dr. Anita Newcomb McGee, of Washington, has just been charged to select women nurses for the army and navy. At first the Government resolved to engage no women nurses, but by the present plan strong, healthy women, between 30 and 50 years of age, having no family ties, and members of training schools of repute, will be drafted for service. The pay is 50 dollars monthly and uniform, which consists of white dress, cap, and apron. They will wear a badge, consisting of a red cross set in a circle of blue enamel. Rations and lodgings will be provided. No nurse will be sent to Cuba, nor permitted upon the men-of-war. There is a great demand for nurses immune to yellow fever, but none have as yet applied.

### OUR QUEEN'S NURSES.

Everyone knowing anything of the work of Queen Victoria's Jubilee nurses must rejoice that a permanent committee has been formed to annually devise some scheme by which those connected with the London and provincial music-halls may aid the association. The Duke of Westminster, who took the chair at a meeting called at Grosvenor House on June 10th to consider the subject, said that the amount subscribed in connection with the Queen's Jubilee Institute had reached the large sum of £156,000, and, after naming some of the sources from which it had been derived, moved the resolution to "form a permanent committee to assist the Queen's Jubilee Nurses' Institute." Lord Compton supported it, and alluded to the national sentiment attaching to the institute, as it was the Queen's own, and she appointed the council.

### WATERFORD INFIRMARY.

The Waterford City and County Infirmary, which has recently been opened, inaugurates a novelty in the training of lady nurses. They are to be under instruction for one and a half years, and to pay an inclusive fee of £25, and to provide uniform. Mrs. Ronayne is the matron, and a large number of applications from candidates have been already received.

### EXPELLED FROM CUBA.

Amongst the number of persons expelled from Cuba on board H.M.S. *Talbot* is Sister Mary Wilberforce, of the British Red Cross Society. It will be interesting to hear the details of the case, as nurses under the Red Cross are regarded as neutral.

### TRAINING CHILDREN'S NURSES.

The Liverpool Ladies' Sanitary Association is promoting a scheme to train children's nurses in the same way that the Norland Institute is managed. Students will be received at 8, Sandon Terrace for a year's instruction. The cost is estimated at rather less than £20.

### THE INDIAN NURSE.

A correspondent of the *Times of Bombay* calls attention to the case of the India-trained nurse, which certainly will command the sympathy of all who love justice. Though all honour be due to devoted women who carry out many an arduous duty away from fatherland and kin amongst strange people speaking unknown tongues, yet equal honour is likewise due to those who unflinchingly meet the daily task that lies nearest in monotonous obscurity. The Indian trained nurses have not failed in their duty. With the experience of years, with full knowledge of the people and their language, they have ministered faithfully to the plague and disease stricken. Amid the flourish of trumpets, with which unfortunately it is all too common for nurses to perform any of the more important undertakings, let not the meed of praise be withheld from the nurses, hard worked and poorly paid, trained in the larger hospitals of our Indian Empire.

### PAROCHIAL SUBSIDIES.

The Local Government Board permits parochial bodies to make money grants to societies for nursing services rendered to those who in times of stress would come upon the rates. The attitude of the Kidderminster Guardians, therefore, towards the District Nursing Associations suggests thought. Mr. Beakbane, the chairman, in proposing the subsidy to the local societies, urged every point in their favour, namely, that they did work that the union ought to do if possible, as half the patients nursed were too poor to pay for themselves; that many patients were enabled by their means to keep out of the infirmary because they could be nursed at home; that others were tided over a difficult strait without appealing for help; and that others, again, received relief for a much shorter time through the administrations of the district nurse. In spite of this excellent case, ably stated, the general opinion was that it was cheaper to engage and pay a nurse for serious cases than to open a door to applications for public money, one gentleman pointing out that before they could be satisfied with such applications a good deal of investigation would be necessary. In reading the account of these proceedings three questions present themselves. Is it not the first duty of Guardians, wisely, zealously, and economically, to care for the poor? Do they fulfil this duty wisely, zealously, or economically by ignoring all the multitude of lesser maladies that the flesh is heir to? And when an enlightened public opinion declares that it is wise and economical to have the poor well nursed, are the Guardians of Kidderminster its fitting representatives?

### DISTRICT NURSING IN ARMAGH.

A GENERAL committee meeting of the Armagh Nursing Association closed a prosperous year's progress of this association. Not only has the income been adequate, but the nurses live rent free by the kindness of Mr. G. A. Edwardes, J.P., and a donation of £50 from J. B. Lonsdale has supplied the furniture. The nurses paid an average of nine visits a day, and 150 cases received attention during the year.

### RUTHIN DISTRICT NURSING ASSOCIATION.

WHEN the subject of engaging a district nurse for Ruthin was first mooted the prevalent opinion was that the expense could not be met. Now, as was stated at the annual meeting recently held at Ruthin Castle (Colonel Cornwallis-West presiding), not only has the money been forthcoming, but considerable developments of the original scheme are contemplated. It is desirable that a second nurse, chiefly for maternity work, should be engaged, and that a nurses' home for the Vale of Clwyd should be established, as there are no nearer centres for supplying nurses than Chester or Liverpool. It was proposed moreover, in the event of the second nurse being appointed, each patient who could afford to employ her should pay, and that the Guardians should be asked to become responsible for those who could not; also, that Mrs. Cornwallis-West and other ladies resident in the neighbourhood, who are much interested in the association, should endeavour to induce the poorer people to join a provident society, which should be managed much in the same way as a clothing club.

### SHORT-TIME CERTIFICATES AT GUY'S.

THE House Committee of Guy's Hospital have decided to give all nurses having to leave the hospital before the completion of their three years' course a certificate stating the time they have been under instruction. This new regulation will prove of much service to such as unfortunately have to curtail the time spent in training; but the greatest care will have to be exercised to prevent confusion between those who hold these new "certificates" and nurses who are fully-trained.

### WORKING LADIES' GUILD.

BY the kindness of the family of the late Sir John Millais the beautiful work of the Working Ladies' Guild was displayed in the studio and drawing-room of his house in Palace Gate. The Duchess of Albany opened the sale at noon on Wednesday, June 8th. Music was provided by Miss May Stevenson's band on the 8th and 10th, and by Miss Bacon's guitar and mandoline band on the 9th. The guild has adopted an excellent way of pricing the articles for disposal, and charging the same rates as the leading trade houses, anything a little out of the common being kindly valued for them by the manager of a well-known establishment. Part of a trousseau was exhibited, the excellence of the material, finish, and cut of which left nothing to be desired. Plain work and children's garments were plentiful, and reasonable in price. There were also stalls of Ceylonese jewellery, the gift of Lady Constance Shaw-Lefevre; art pottery from Weymes and Thun, poker work, and church embroidery, thus giving the purchasers a varied choice. Ladies desiring trousseaux or layettes will find many novelties at the show-room of the Guild, 251, Brompton Road, S.W., and whilst they pay only the market value for these things they help a class that suffers very keenly from the pangs of poverty, namely, the poor gentlefolk.

### SCARCITY OF NURSES IN CALCUTTA.

THE committee of the Calcutta Hospital Nurses' Institution have written to the editor of the *Englishman*, making the following statements. The primary object of the institution is to provide nurses to attend the poorer classes of Europeans and natives who are patients in the European General, the Eden, and the Medical College Hospitals. Additions of four out-nurses within as many years have been made, but the supply is not nearly equal to the demand, and until the new hospital is built or the nurses' home enlarged there is no room to accommodate any more. Whilst no case, therefore, is refused when there is a nurse to spare, it is impossible to comply with all requirements. Calcutta has also the eight nurses of the Canning Home, but as they are generally engaged months ahead, there is much difficulty in getting help in an emergency. The subscribers to the Calcutta Hospital Nurses' Institution instead of complaining of the dearth of nurses, and threatening to withdraw their subscriptions from the institution, should see that more money is needed to bring up the nursing service to the needs of the city.

### SHORT ITEMS.

MISS M. SAUNDERSON has been appointed superintendent nurse of the Hull Workhouse at a salary of £40 a year, subjected to annual increments until £50 is reached.—Mrs. Dickinson Berry, who has recently been appointed to examine the "defective" children for the London School Board, studied at the London School of Medicine for Women and took the degrees M.D. and B.S. She also held some important posts at the New Hospital for Women, Euston Road.—A branch of the *Hamadryad* Hospital Ship has been established by the Barry Nursing and Accident Hospital.—It is resolved to build increased accommodation for the nurses and servants of the Aberdeen City Hospital at a cost of £4,650.—The salaries of charge nurses at Islington Workhouse are in future to begin at £24 and to increase to £28 a year by a £2 annual rise.—A pathetic interest is attached to Miss Wynne's ("Sister Maude") untimely death at Cork Fever Hospital. A fellow-nurse, when stricken with typhus, besought the sister to nurse her herself, and was safely brought to convalescence. Miss Wynne, however, unfortunately contracted the fatal disease and died.—A fire broke out between two and three a.m. on the morning of May 29th in a block of outbuildings at the Mary Wardell Convalescent Home, Stanmore, Middlesex, by which a gardener's potting-house and two servants' bedrooms were completely destroyed.—Miss Dunn, superintendent of the Queen Victoria Jubilee Nurses in Ireland, is at present taking leave, after great family bereavement, and is gone abroad; Miss Blackman, her assistant and secretary, is in authority meantime, at the offices, 14, Nassau Street.

### Post-Graduate Clinics for Nurses.

By A Trained Nurse.

### ARTIFICIAL METHODS OF FEEDING.

### Rectal Feeding.—III.

In rectal feeding the nurse should always take advantage of a natural motion, and should give a nutrient enema very soon after this has taken place. Many doctors prescribe with rectal feeding the addition of an antiseptic to the daily washing-out enema. Boracic is the most commonly used, and serves not only as a general sweetener, but assists in preventing the fermentation of the products of rectal digestion. The daily soap and water injection washes away the waste matter of previous nutriment in addition to acting as a general intestinal tonic.

In hæmorrhoidal conditions, and when much anal irritation is present, the doctor may entrust the nurse with a solution of cocaine to be applied locally before each injection. The addition of some preparation of opium to the nutrient is common, and this is given to soothe and to allay peristalsis. A newer and more favourite method, however, consists in injecting the opium preparation by itself about forty minutes before a nutrient enema is due. Sometimes, however, an opium suppository is preferred to this method. Many doctors impress upon the nurse that in beginning the use of nutrient enemata it is best to administer one every five hours, this consisting of from two and a half to three ounces in quantity. As the bowel grows used to the operation—and in skilful hands, this will very likely prove the case—an enema may be given every four hours, and in very favourable cases every three hours. It is a great mistake to administer more than two and a half or three ounces at one time. A larger quantity than this for each injection is liable to cause trouble, and it is far better to be able to administer the enemata constantly than to inject large quantities for two to three days and see the end in failure and diarrhœa.

There is a growing feeling in favour of the peptonisation of all food intended to be injected into the rectum. Milk thickened with cream, eggs, and some starchy food used to be injected without pre-digestion. But later experience seems to be in favour of the pre-digestion even of egg albumen, although some authorities state that the rectum has the power of digesting this absolutely without the aid of artificial means, especially when a small quantity of common salt is added, not more than 10 grains to each egg used. When irritability of the bowel exists barley will be found of infinite value. Milk for rectal feeding must not be diluted with barley water, for where so little nourishment is available this must be as concentrated as possible. Barley milk containing all the nourishment of milk with the soothing qualities of barley, and subsequently peptonised, will be found most valuable. In giving alcohol by the rectum many doctors prefer that this should not be mixed with food, as they say it is apt, given thus, to finally and soon disagree. Mixed with barley water and injected separately, it seems to be retained better and to serve a more useful purpose. A little milk-sugar may form a useful nutritive factor in the enema.

Fatty substances appear to have very little value in rectal feeding. Fats are said to coat the mucous membrane, and so prevent the absorption of other substances, and yolk of egg is not recommended because of the fatty matter contained in this. Good milk pancreatised with fresh raw meat juice and white of egg is an admirable composition. The doctor should be consulted as to the addition of salt to the egg albumen, for though this assists its digestion it needs to be used with caution, since it is liable to cause irritation.

The main object in the pre-digestion of rectal foods is to prevent waste matter resulting from digestion accumulating in the bowel. Any food which leaves much residue is therefore to be avoided, since this is very liable to set up an irritation which soon leads to diarrhœa. Meat peptone is much used for rectal nutriment, and proves most valuable, but the nurse must take care not to give this in too concentrated a form, for if so it will be almost sure to set up irritability. Meat peptone one part, barley milk two parts, proves an excellent formula which is usually well retained. Cream used to be largely employed in nutrient enemata, but has fallen much out of favour, since it is included among the fats which tend to coat other food and the mucous membrane itself, and so prevent their absorption by the bowel. Malt extracts are a very useful addition to enemata, since the form of nutriment contained in such preparations can be assimilated by the bowel, and such extracts are soothing and bland in substance. Pancreatised milk thickened with Mellin's or Benger's Food, as well as varieties of pancreatised gruels may be used with advantage, and two teaspoons of Valentine's meat juice included in any of the nutrient enemata will add a valuable factor of nourishment. This meat juice given with the whites of two eggs will make up a very satisfactory enema.

I have already pointed out that many doctors prefer alcohol to be given as a separate injection, and the reason for this is that spirit added even to pre-digested milk sometimes causes that casein which has not been converted into peptone to be coagulated, and this coagulated matter is liable to cause irritation in the bowel. Mixing with barley water counteracts the tendency of alcohol to cause irritation, while two to three teaspoons of malt extract will not only add to the thickness of this enema, but will give a valuable nutritive quality.

A few words now as to the quantity of food for the twenty-four hours. This must depend, first, on how often the injections can be borne and on the age of the patient. In an adult, if the injection is given only once in five hours, I should give in each enema the whites of two eggs and should make up the necessary quantity by using alternately formulæ like the following: Two whites of egg, with or without ten grains of salt to each egg, this according to doctor's instructions. Make up to two and-a-half or three ounces by adding peptonised milk gruel and two teaspoons of Valentine's meat juice. For the succeeding enema I would take two whites of egg, and would complete the desired quantity by fresh raw meat juice and two teaspoons of malt extract. The next enema might still have the white of egg basis, to which I would add beef peptone diluted with two parts of barley water or barley milk. Under this *régime*, if the injections are given with all the precautions previously laid down, the patient ought to do very well.

Some doctors use Leube's method of feeding by the rectum, and it is important that the private nurse should understand how to prepare an injection by this formula. Fresh pancreas is added to lean beef in the proportion of one part of the former to three of the beef. Both should be so finely scraped that when rubbed up with a little warm water a fine paste is readily formed, which, either by itself or mixed with white of egg, is then injected by means of a wide rectal tube. The nurse must carefully free the beef from all fat, sinew, &c., before scraping. In going thus thoroughly into the question of rectal feeding I have done so because I believe that every fresh piece of knowledge which a nurse obtains in the science of feeding is of the utmost value to her in gaining professional success. Rectal feeding would not break down so frequently as it does were nurses more thoroughly acquainted with the minutiæ of this very important branch of nursing. Bearing in mind the metal-tubed syringes and the raw cornflour mixed with raw milk which still sometimes does duty for nutrient enemata, I have taken the more interest in presenting the other side of the picture.

### Nursing in Paris Hospitals.

### B.—TRAINING OF LAY NURSES.

### VII.—Outside Scholars.

The Paris schools for nurses are all frequented by a certain number of outside scholars, and, as I have shown in the last two articles, two schools are entirely for outsiders—the school for midwives at La Maternité and the Clinique d'Accouchement.

It is only within the last year or two that the most important element has begun to enter into the general hospital schools, that of women of superior education and position, who wish to arm themselves for the carrying on in private life the noblest of all womanly functions, the aiding the afflicted. For many years almost the only externes in the hospital schools were a sprinkling of sick nurses who wished to perfect themselves sufficiently to be worthy of recommendation for employment by physicians and surgeons. These were, for a number of years, chiefly found at La Pitié and La Salpêtrière, there being at La Pitié in 1891, 16 externes (two male and 14 females), of whom 13 were given certificates. The next year at La Salpêtrière were 17 externes in the school.

With the establishment of Lariboisière school a new element was infused in the class of externes. The number of these soon exceeded by more than double that at any of the other schools. The number of externes in 1895 was only 10 (five of each sex), but increased to 17 (all women) in 1896, and to 45 in 1897, of which last number 22 women and two men were given their diplomas. The class of Lariboisière externes was noted as including women of refinement, some o whom were members of the famous charitable organisations the Femmes de France and the Dames Françaises, one of the scholars being a full-blown marquise. These new Lariboisière externes seem to make a partial truce in the bitter feud over laicisation, as the Union des Femmes de France and its sister association, the Société des Dames Françaises, are semi-religious bodies, of course strongly in favour of the Sisters, and organisers of rival systems of succour to the sick and afflicted. They have each had heretofore private schools of their own, only using the hospitals for practical exercises, and the administrative sanction for this has been a strong grievance with the laic champions, because similar privileges were not granted to the outside pupils in the hospital schools.

Of course, the bulk of the outside scholars are private sick nurses, who are gradually drifting more and more into the hospital schools. Hitherto the larger number of this class in Paris had no special training at all, and the minority who have had any benefit of nursing schools have been attendants at the before-mentioned courses, organised by the Femmes de France and the Dames Françaises, or similar courses instituted by the Red Cross Society (Société Française de Secours aux Blessés) or the Policlinique de Paris. Every effort is made by the friends of the hospital schools to attract this class of students, and at La Pitié and La Salpêtrière with some show of success. Last year at the former were 22 externes (20 women and two men), of whom 16 (15 women and one man) were given certificates, and at the latter 12 certificates were allotted to externes.

As an inducement to sick nurses to attend the schools it is proposed to keep at the hospitals employment registers for all the certificated nurses to enter their names when desiring an engagement. As yet the administration has only consented to such a register at La Pitié, where the largest number of externes has as yet been certificated. Doubtless at Lariboisière, which now comes to the front as a favourite of the externes, another register will soon be set up. Of course, the register is free for both employer and employed. As anyone in Paris can set up as a private sick nurse, with no qualifications at all, this spreading of opportunity for special qualification is most timely.

Yet another proposition for attracting the outside sick nurses to the hospitals for instruction is the offer of facilities for accompanying the hospital doctors and surgeons in their private visits for experience and instruction. It is not so put forth, but doubtless there is underlying this the notion that by such a round of visits in private houses the sick nurse is likely to be put in the way of many private engagements.

Another class of semi-outside students, one which I have once or twice before briefly alluded to, remains to be mentioned. This is those constant sources of contention in the hospital administration, the boursières, or holders of municipal scholarships. These were originally instituted, as before stated, when material to replace the sisters at laicisation was deficient, and special inducements had to be offered for expert services. The boursières are selected from outside by one of the committees of the Municipal Council, which votes special funds for the payment of the scholarships. The candidates are usually of a superior class and well educated. They receive 1,000 francs a year, but have to live outside the hospital. On appointment they take the position of ordinary nurse, and at the end of the year, if they get their certificates, are appointed matrons or superintendents, and assume all the duties of such offices. The great complaints against them are that they will not do the ordinary work of probationers or assistant nurses, considering themselves above it. Moreover, it is alleged that the hospital directors have no control over them, nominated as they are by the Municipal Council, and the directors are unable to discharge them in case either of negligence or disobedience. It is also, of course, a natural complaint that at the end of the year they get promoted over the heads of the regular first-class nurses (a class they are able to skip), nurses who have often held certificates for three or four years. Hence it is that the boursières are the pests of the house doctors and surgeons. Furthermore, it is said that there is much political and personal favouritism in the nominations. Thus it is that the boursières are reckoned by the hospital staff, from the top to the bottom, as rank outsiders in many senses.

Edmund R. Spearman.

### Presentations.

The district nurse of Baledgarno, Inchture, Miss Douglas, was presented on June 9th with a handsome dressing case and a purse of sovereigns, on the occasion of her quitting the neighbourhood. Miss Douglas has been engaged by the Jubilee Nurses at Edinburgh, and leaves many friends behind her.

### The Care of the Feeble-Minded.

On Friday last the Duchess of Sutherland presided over an afternoon meeting at Stafford House to consider and adopt resolutions to the effect that the condition of the feeble-minded called for immediate attention; to ensure it by forming a national association for promoting their welfare; and to urge forward necessary legislative measures. The Duchess of Sutherland, who is also the president of the association, gave a clear outline of the state of the feeble-minded, and said that the society came before the public with definite aims. The State recognised as mentally deficient only idiots and lunatics, ignoring the large number who fall below the normal standard, and who under skilled training can be redeemed from misery and from becoming a source of danger and menace to society. The first aim of the new association is to increase the number of small homes, which have been proved so valuable; to board out such children as are best suited to that mode of treatment; to supply specially trained teachers; and to establish laundries and other industries by which those who require discipline may maintain themselves either partially or wholly.

The first resolution, proposed by Lord Herschell, seconded by Sir Douglas Galton, F.R.S., and supported by Sir James Crichton Browne, ran thus: "That in the opinion of this meeting the existence of large classes of feeble-minded persons is a serious danger to the moral and physical welfare of the country, and calls for immediate attention both on the part of public authorities and charitable enterprise."

Lord Herschell said that the new society stood on its defence and needed justification. The evil it was designed to contend against was no new one, but the recognition and classification of the different grades of feeble-mindedness had only recently been studied. The mentally deficient were heavily handicapped by nature in the struggle for existence, and they were helpless and their condition hopeless unless equipped by the best training. Their condition under training was hopeful, their laggard intellect could be aroused, and those apparently incapable of work might be shown to be capable of most excellent work. He pointed out that on public grounds the care of the feeble-minded was a duty devolving on the nation, as neglected they drifted into the army of paupers and criminals chargeable on the State.

Sir Douglas Galton insisted that justice required the classification and training of those of weaker intellect, one-half of whom, under proper instruction, become self-supporting, one-third partially so, and one-sixth only remaining an entire charge.

Sir James Crichton Browne said that the feeble-minded were a source of danger and contamination drifting through the world in a state of semi-starvation, gorging the workhouses, thronging the hospitals, crowding the prisons. Yet the masses of human waste were individuals—the burden and curse of their own generation, they bequeathed burdens and curses to the generations to come. As a body they were pitiable, they were much more sentient than idiots, pathetically conscious that they were different from others, and feeling their inferiority keenly. As a rule, they were amiable and well disposed. Their rescue has been undertaken and carried on by societies doing valuable work in arousing public attention. He estimated that there were 23,000 and more weak-minded persons scattered amongst the population, a number likely to be increased in the immediate future. We prided ourselves on the reduction in the death-rate. The decrease, however, has not been in the lives of the mature, but in infants under five. We succeeded in prolonging feeble lives, curtailing the ravages of bacilli, to increase our own burdens. The grinding toil and insufficient nourishment of the poorest in our civilisation did not promote brain health, and acted still more injuriously on their offspring, whilst the excitement and surfeit of the wealthy were even better manufactories of mental deficiency. Feebleness of mind was, he said, often curable. The motor centres of the brain not only regulated the muscles, but regular exercise of the muscles invigorated and increased the motor centres, giving wider powers of mental attainment. He recommended industrial training and discipline.

The second resolution was proposed by Mrs. Garrett Anderson, M.D., and seconded by Mr. W. H. Dickinson, "That the attempt which is being made to deal with this subject by the National Association for Promoting the Welfare of the Feeble-minded is one that deserves generous public support."

Mrs. Garrett Anderson, M.D., confined her remarks to practical ways of raising money. She suggested that the parents of healthy and good children should make a thank-offering to the funds of the society on the birthdays of their children, say, 5s. for character and 5s. for brain power. They could begin as young as possible, three months was old enough to show a child's mental capacity, and a baby of a year old had reached quite an advanced age.

Mr. W. H. Dickinson, chairman of the Executive Committee, gave a sketch of the rise of the society, the number of cases applying for help, their need of £1,000 yearly for five years, and the expenses incurred on behalf of each individual cared for. The cost was 10s. a week, but as the relatives or parish guardians contributed 7s. a week, only 3s. a week per case was needed from charity.

The third resolution, proposed by Sir U. J. Kay-Shuttleworth, Bart., was "That, in the opinion of this meeting, the condition of the feeble-minded requires the early attention of Parliament with a view to special legislation on the subject."

Miss Dendy, in seconding, gave particulars of her own experience in dealing with the feeble-minded.

The meeting, which adopted the resolutions unanimously, terminated upon passing a vote of thanks to the Duchess of Sutherland. The office of the National Association for Promoting the Welfare of the Feeble-minded is 49, Victoria Street, S.W.

### Physical Exercises at the Queen's Hall.

On June 11th, at three p.m., a charming entertainment was given at the Queen's Hall, Langham Place, by the girls of the North Hackney High School, in order to raise the money necessary to support a nurse, whose duty it will be to visit the elementary schools in poor districts in their neighbourhood, as proposed by Miss Honnor Morten. In addition to the juvenile performers, Miss Mary Owen, Miss Fanny Kreuz, and the Amphion Glee-men contributed an attractive selection of songs, Mr. Fountain Meen gave some organ solos, and the Northfield House String Orchestra a selection of pieces.

The young ladies appeared first in dumb-bell exercises. They were dressed in kilted skirts and jersey bodices of dark blue, with crimson collar and sashes. The second series of exercises were arranged with large wooden rings, the third with balls, and the last with cymbals. They wore in this white Greek costumes, and the display was very graceful and picturesque, evoking hearty applause. Miss James, their instructress, herself directed the exercises, which seem admirably adapted for the results desired, her pupils showing themselves light and active in their movements. Messrs. Macmillan—who announce that Miss James' book on "Girls' Physical Training" is now ready—generously printed the programme free of charge. The entertainment realised about £50, enough to supply one school with a nurse.

### In Memoriam.

WE are sure our readers will pardon any repetition of information relating to the deaths of Sister Frances and Sister Gertrude already given in the "Mirror," for the sake of the fuller details just received from Hong Kong.

Sister Frances was a daughter of Mr. Higgin, of Carrick Fergus, Ireland. She entered Guy's Hospital in 1887, and after completing her training she went to the Marylebone Infirmary as sister, and from there came out to Hong Kong in 1890, being amongst the first trained nursing sisters introduced into the Government Civil Hospital. She went through the plague epidemic of 1894, and proceeded home on leave in 1896, returning to the colony early in 1897 as keen as ever on her work. She was nursing a patient, one of the ward boys, who in his delirium coughed in her face, and four days after she complained of being ill. It was soon realised that she had contracted the disease in its worst form —pneumonic plague—and in spite of all care on the part of her fellow sisters she rapidly succumbed, and died on April 29th. She was buried in the Happy Valley Cemetery, in a spot chosen by herself only a short time before, attended to her last resting place by representatives of all sections of the community. Coming in contact with patients of all classes and nationalities, by whom she was much beloved, she had ample opportunity of proving her sterling worth, and her loss will be keenly felt by all those who had the high privilege of coming under her care. Socially she will be equally missed, for her cheery presence was well known here and her kind and generous help always available in the cause of charity. It seems difficult to offer much consolation to those who are left to mourn her loss, but she will not have sacrificed her life in vain if she has helped to instil into those left behind the great necessity of doing one's duty without fear or trembling.

Hardly had the earth grown cold on Sister Frances' grave before the staff of the Government Civil Hospital, Hong Kong, were called upon to bear another heavy loss from plague in the person of Sister Gertrude. She, unfortunately, contracted the disease in her devoted attendance on Sister Frances, and on May 2nd first complained of being ill. She was at once warded, and, in spite of every care and attention possible, succumbed to pneumonic plague early on the morning of the 5th, conscious almost to the last. Sister Gertrude was the daughter of Mr. Ireland, of Salle Repham, Norfolk, and began her training at St. Pancras Infirmary, subsequently going for further instruction to the London Hospital in 1886. She served some time at the latter institution as "night sister" and "Sister Blizzard," coming out to Hong Kong in 1890 to inaugurate the new era of trained nurses. Like Sister Frances, she went all through the first epidemic of plague, and then proceeded home on leave. On her return she acted as matron during the absence of Miss Eastmond, and had only just returned to her usual work of ward sister. She was buried close to Sister Frances, accompanied to the grave by a large gathering, all anxious to pay respect to one who had so fearlessly laid down her life. At their own special request, the European section of the police, amongst whom were many of her former patients, provided an escort and carried the coffin from the hospital to its last resting-place. This exceptional mark of respect was deeply appreciated by her fellow-sisters and the staff. Gifted with a bright and cheery smile and a most winning manner, she made an ideal nurse, and to many of her patients it must have seemed almost a pleasure to be ill under her care. Ever ready with kindly advice and unfailing sympathy to the staff, her loss is indeed a blow which seems as if even Time itself would never heal. Those in trouble found in her an ideal comforter, and it seems hard to realise that never again shall we see her cheery smile or hear her winning voice.

### Everybody's Opinion.

[Correspondence on all subjects is invited, but we cannot in any way be responsible for the opinions expressed by our correspondents. No communication can be entertained if the name and address of the correspondent is not given, as a guarantee of good faith but not necessarily for publication, or unless one side of the paper only is written on.]

### NURSING IN CAIRO.

"H. C. F." writes: I see in last week's HOSPITAL that the correctness of the information I sent you about nursing in Cairo has been questioned by "H. T." I would like to say that the information I sent had been received by me direct from Cairo from a member of the nursing staff of the Kasr-el-aini Hospital, from whose letter I quoted when writing to you; it must therefore be quite correct. I know the Kasr-el-aini Hospital is for natives only, and the only error I made in my statement was in adding that I thought the private staff of three nurses was connected with the hospital. If Miss James's private home is not existing it must have been lately abolished.

### FOR THE "LONDON."

MISS MAULEY writes: Will you kindly allow us to reach most of our London Hospital friends through your paper? We are sure that all the nurses who have ever enjoyed the privileges of the "London" want to help the hospital to the best of their ability. A fund has been opened in aid of the hospital, and all "Old Londoners" are invited to join. The subscription is 2s. 6d., to be sent before August 10th, 1898, to Miss Mauley, Foston, Park Lane, Croydon. The following ladies have subscribed their names to this letter: Miss Mauley, W. Russell, E. H. Potts, J. E. Collinson, H. Hetherington, E. Yeates, and E. J. Moir.

[*** We gladly accede to our correspondent's request, and have pleasure in thus affording to all our readers who were trained at, or who are for other reasons interested in, this hospital an opportunity of contributing to the fund now being raised to establish its financial position.—ED. *T. H.*]

### NURSING IN WORKHOUSES ORDER, 1897.

"PHYLLIS" writes: I read so much likely to prejudice nurses against this new opening that I send my experience of it. I am in my first post as superintendent nurse, being also the first appointed here. From the Guardians I have received only courtesy, from the "untrained" matron much kindness and valuable advice, possibly because I have from the first recognised her authority in all matters except nursing and consulted her and the master before making alterations. It seems clear to me from the Order that this is the way we are meant to work and reform. And in all matters concerning the treatment of the sick the medical officer must be consulted and obeyed, or if he is a busy non-resident man pains taken to get into his "ways" and so gain his general approval. The nurse who fails to win the confidence of these "powers that be" may as well resign, the patients will soon perceive the state of affairs and take advantage of it. I strongly advise hospital-trained nurses who are thinking of taking these posts to train in midwifery, or if already qualified in that take a post as charge nurse in one of the larger infirmaries, thus gaining knowledge of the most possible to be done under the Poor Law and so avoid many mistakes. For myself I find it a happy, busy, and, I hope, useful life, with quite as much power as is good for me, the wear and tear much less than in hospital, the calls on my private purse less. Difficulties and inconveniences are to be attacked gradually. I know from personal experience that my patients are better nursed than is possible in many ratepayers' homes, therefore make the best of what is provided and ask only for absolute necessities. Nurses need to realise that Boards of Guardians, and even masters and matrons, are generally trying to do their duty from their point of view, which they are not accustomed to change quickly, and when they find the nurse does hers they will listen to her suggestions for the greater comfort of the patients or for labour-saving appliances

### Nursing in India.

### I.—NOTES ON A CHOLERA SEASON.

I was roused from sleep at a quarter to twelve p.m. by the night nurse on duty, who said that the civil surgeon was waiting to take me to a cholera case. A lady in a saloon carriage at the station had been attacked with cholera. I was over at the hospital entrance in a few minutes, and found that the doctor had collected the few necessaries, namely, our stock cholera mixture, hypodermic syringe, ether and digitalis, &c. On our way to the station the doctor said he thought it would be better if I could keep the patient in ignorance of the fact that she was suffering from cholera. On my arrival I found her suffering from severe cramp in the extremities, marked restlessness, covered with cold clammy perspiration, pulse very feeble, and incessant vomiting and purging, and overpowering thirst. I at once procured hot water bottles, and kept up gentle friction until I could go on no longer, then I applied mustard plasters to the calves of the legs, and to the muscles of the upper arm. I found the patient had taken nothing but Brand's essence and brandy. We were able, through the kindness of the station officials, to procure some good milk, and abundance of ice. The cholera mixture I gave every second hour, and at four a.m. gave her an injection of ether and digitalis, as the radial pulse showed signs of failure. It was a terrible night that we passed in the saloon carriage, with the patient tossing restlessly on the narrow seats.

About five a.m. the cramps subsided in a slight degree, and the patient was able to keep down feeds of ʒii of iced milk, with brandy. The profuse perspiration continued, but the purging had become less violent.

At nine a.m. the "dholie" arrived to convey my case to our own cholera ward. We "cupped" over the kidneys every second hour, fomented, kept up friction, hot-water bottles, &c., and though the patient retained the small iced feeds and stimulant, we were obliged to have the medicine omitted, as it appeared to produce troublesome vomiting. After thirty-eight hours of total suppression, the patient passed a few drops of urine, and from that time made rapid progress, so far as her choleraic condition was concerned, but serious nervous debility followed, lasting for some months. I was one of her "specials" for about a fortnight, when another case came in, and I left the patient to a regular day nurse. She suffered from severe dyspepsia for a long time after she was discharged from hospital, but eventually a long rest in Simla set her quite up.

The peculiar change in the colour of the motions in all cholera cases is worth noting. In those which terminate favourably I have often seen the typical "rice-water" stool succeeded by one of a very pale yellowish-pink. Gradually the motions seem to consist of nothing but bile until the reaction is safely past and perfectly normal evacuations take place. In cases terminating fatally the motions change from the "rice-water" to a port wine colour, closely resembling the hæmorrhagic motions of an enteric. I have never seen a case recover after this change in the evacuations has taken place.

"Fakirs" (begging priests) are difficult patients to deal with at all times, but as cholera cases they are hopelessly unmanageable. I had one last year. He was an opium eater, and had been used to keeping it in his hair, which was supplemented, as is the rule with fakirs, by coils of rope, and these coils had fallen down in his restlessness, and, his opium being lost, he became quite frantic.

Attached to each ward is a large bath-room, with a tap; one day the intermediate door had by some oversight been left unbolted. I went into the ward, and to my astonishment the bed was empty. I heard the tap running and went in, to find my patient on his back on the floor, his head just under the tap, which was turned on full, and the water simply flowing down his throat. I called help, and we carried the poor "fakir" back to bed. I must tell you that this was one of those cases in which all secretions had stopped, even the profuse perspiration, so that the quantity of water the patient had swallowed had no escape. He swelled up till he resembled a drowned case which has been long in the water. Needless to say, he died that same evening.

We had sixty-four deaths and thirty-four recoveries while I was in charge of the ward. The rains and cholera always come together, and this makes it most difficult to get the ward linen dried, and the rainy season tends to make both nurse and patient much depressed at times, but during a cholera season the nurses are too busy to give way to feelings of that sort.

### The Matrons' Council.

On June 15th and 16th the Matrons' Council held its first annual session at the rooms of the Medical Society, 11, Chandos Street, Cavendish Square. Miss Isla Stewart, matron of St. Bartholomew's Hospital, took the chair, and about forty were present at the morning meeting, when papers were read by Miss Mollett, matron of the Royal South Hants Infirmary, on "The Matron's Duty to Her Profession," and by Miss Elinor Pell-Smith, lady superintendent of the Home Hospital, Leicester. In the afternoon Miss Smith's paper on "The Training of Male Nurses" was read by Mrs. Bedford Fenwick, who supplemented it by some notes on training army orderlies, by an army nursing sister of ten years' experience. The discussion following this paper was rendered particularly interesting by the comments and information of Mr. Walsh, the principal of the Male Nurses' Co-operation.

### Appointments.

### MATRONS.

The Government Civil Hospital, Hong Kong.—Miss Helen Batchelor has been appointed by the Colonial Office to the post of Sister of this hospital, and sailed to take up her new appointment on the 11th inst. She rec ived her training at the London Hospital (1894 to 1897), and took the L.O.S. certificate after sp cial training at the Clapham Maternity Home. She then became lady superintendent of St. John's Hospital, Winchester, and subsequently fulfilled the duties of temporary matron and secretary at the Herne Bay Convalescent Home.

Chesterfield and North Derbyshire Hospital and Dispensary.—Miss Annie Scott was elected Matron of this institution on the 9th inst. She was trained and afterwards charge nurse at University College Hospital, where she was also housekeeper of the Nurses' Home. She then successively became head nurse of the Stockton-on-Tees and Thorsley District Nursing Association, superintendent nurse of Hickleton Main Colly Accident and District Nursing Association, and night sister superintendent at St. John's Infirmary, Hampstead, London.

Llanelly Hospital.—Miss L. A. Roberts has been appointed Nurse-Matron of this hospital. She was trained at the Hope Infirmary, Manchester, and has been since nurse at Bishops Stortford Infirmary, and, later, at the Northern Counties Hospital, Manchester, and charge nurse at Craigleith Hospital, Edinburgh.

### Minor Appointments.

Poole Union Workhouse.—Miss Elizabeth Shaw has been appointed Superintendent Nurse of this workhouse. She was trained at Chorlton Union Hospital, Withington, near Manchester, and has previously held positions in Rochdale Union, Toxteth Park Infirmary, Liverpool, and latterly she has been engaged in private nursing.

### For Reading to the Sick.

### "FRET NOT THYSELF."

**Verses.**

I SOUGHT for Peace, but could not find;
I sought it in the city,
But they were of another mind,—
The more's the pity.

I sought for Peace of country swain,
And yet I could not find;
So I, returning home again,
Left Peace behind.

Sweet Peace, where dost thou dwell? said I—
Methought a voice was given,
"Peace dwells not here, long since did fly
To God in Heaven."

Thought I, this echo is but vain,

To folly 'tis of kin;

Anon I heard it tell me plain,

'Twas killed by sin.

Then I believed the former voice,
And rested well content;
Laid down and slept, rose, did rejoice,
And then to Heaven went,
There I inquired for Peace, and found it true—
An Heavenly plant it was, and sweetly grew.
—*Anon.*

How strange that all
The terrors, pains, and early miseries,
Regrets, vexations, lassitudes interfused
Within my mind, should e'er have borne a part
(And that a needful part) in waking up
That calm existence which is mine,—when I
Am worthy of myself. —*Wordsworth.*

I do not ask, O Lord, that Thou should'st shed
Full radiance here;
Give but a ray of Peace that I may tread
Without a fear!
Joy is like a restless day; but Peace divine
Like quiet Night;
Lead me, O Lord, till perfect Day shall shine
Through Peace to Light! —*A. Procter.*

Grant us Thy Peace throughout our earthly life,
Our balm in sorrow, and our stay in strife;
Then, when Thy voice shall bid our conflict cease,
Call us, O Lord, to Thine eternal Peace. —*Ellerton.*

**Reading.**

The lintel-stones and pillars of the New Jerusalem suffer more knocks of God's hammer and tools than the common and side-wall stones. And if twenty crosses be written for you in God's Book they will come to nineteen and then at last to one, and after that nothing but Christ's own soft Hands to dry your face and wipe away your tears.

It is much to come out of the Lord's school of trial wiser and more experienced in the ways of God. We cannot of ourselves take away the base metal that remaineth in us; and if Christ be not Master of work, and if He were not standing nigh the melting of His own vessel, the labour were lost and the founder would melt in vain. God knoweth some of us have caused the loss of much fire and pains to our Lord Jesus. Some of us must answer to the Majesty of God for the abuse of many good crosses, and rich afflictions lost without the quiet fruit of righteousness.

What God layeth on let us suffer; for some have one cross, some seven, some ten, some half a cross. Yet all the Saints have whole and full joy, and seven crosses have seven joys.

But if our ill-health is wounding to our pride, let us face the fact and accept it as a link which binds us all the closer to Him who went through dishonoured suffering. The Cross has so long been a sign of victory that we forget the social ignominy of the road to Calvary.—From *Stray Thoughts for Invalids.*

### Notes and Queries.

**The contents of the Editor's Letter-box have now reached such unwieldy proportions that it has become necessary to establish a hard and fast rule regarding Answers to Correspondents. In future, all questions requiring replies will continue to be answered in this column without any fee. If an answer is required by letter, a fee of half-a-crown must be enclosed with the note containing the enquiry. We are always pleased to help our numerous correspondents to the fullest extent, and we can trust them to sympathise in the overwhelming amount of writing which makes the new rules a necessity. Every communication must be accompanied by the writer's name and address, otherwise it will receive no attention.**

*L.O.S.*

(108) Kindly inform me of the best way in which to obtain the L.O.S. certificate. I have a doctor's certificate witnessing that I have attended thirty cases and lectures.—*Draw.*

You will only need to pass the examination. The Secretary, the Midwife's Institute, 12, Buckingham Street, Strand, would arrange it for you.

Nurse E. H. Thomson draws our attention to the fact that attendance on 20 (and not 25, as stated) cases is sufficient for L.O.S.

*General Training.*

(109) What infirmary or hospital would you advise me to enter for general training? I want a small salary and uniform, and I hold the certificate of the Medico-Psychological Society.—*Nurse Walker.*

You evidently wish to supplement your mental training with that of a sick nurse, therefore it would be advisable to enter any recognised training school and take your three years' certificate, without reference to the mental training you have had. There are numerous training schools where salaries are given.

*Training.*

(110) I should like to become a nurse in a hospital or infirmary. I hold two certificates, one from the St. John Ambulance Association for first aid and one for sick nursing. Should I apply as probationer or assistant nurse?—*S. A. C. (Leeds).*

See Honnor Morten's "How to Become a Nurse" (2s. 6d., post free, from The Scientific Press, London), and apply for admittance as a probationer. Your certificates will not be of any advantage to you, as most institutions prefer to train their pupils in their own way. If you enter a Poor Law infirmary, make sure that it is recognised as a training school by the Local Government Board, in order that your three-year certificate may qualify you for the post of superintendent nurse.

*American Diploma.*

(111) Could you tell me how I could get any nursing to do, either in a hospital or in a nursing home? I have an American diploma, having trained and graduated in a very large general hospital in Philadelphia. Could I obtain a post as substitute during vacation time?—*America.*

We have made inquiries at two of the principal nursing co-operations, and find that although they did not accept American qualifications for their own staffs, yet the superintendent thought that qualifications ranking with the John Hopkins' training would be sufficient for any home. See and answer suitable advertisements in the "Mirror."

*The Victorian Order of Nurses.*

(112) What qualifications are needed by nurses desirous of joining the Victorian Order of Nurses? Would it be an advantage to have the L.O.S. certificate? I intend returning to British Columbia shortly, and will be glad of any information.—*E. D.*

The Secretary, the Victorian Order of Nurses, Ottawa, Canada, will supply you with information. In the meantime some of our readers will perhaps give you information from their personal experience. A thorough knowledge of midwifery is certain to prove useful, if not absolutely necessary, amongst a scattered population.

*Institution for the Feeble-Minded.*

(113) Will you tell me where there is an institution for feeble-minded boys?—*G. E.*

The National Association for the Promotion of the Welfare of the Feeble Minded have their offices at 49, Victoria Street, Westminster. An account of their work appears in this week's "Mirror."

*Massage.*

(114) Will you kindly tell me the names of some good schools for massage, and give me any information that may be useful?—*K. T. S.*

One of the best schools is the National Hospital for the Paralyzed and Epileptic, Queen's Square, Bloomsbury. The Society of Masseuse, 12, Buckingham Street, Strand, gives full information on questions relating to massage.

*The Poor Law Nursing Code.*

(115) In an institution under the Poor Law a code of rules for the staff has been drawn up and printed in book form by the managers. These, for reasons of her own, have been suppressed by the matron, who has only given to each member a single page relating to his or her duty. Would any officer be justified in requesting a copy of the whole book and in refusing extracts from it?—*Verity.*

With regard to the code of rules referred to by "Verity" it would be an unheard-of thing to furnish subordinate members of the staff with a complete copy, containing, among other things, regulations for their superior officers, nor have they any right to expect it. Subordinates are concerned only with those regulations relating to themselves, of which the matron appears to have furnished them with a copy. The duties of the officers, as a whole, concern the heads of the establishment, who alone are responsible to the managers for the efficiency with which those duties are carried out.

